# Relation Between Wire Resistance and Fluid Pressure in the Transient Hot-Wire Method

**DOI:** 10.6028/jres.094.014

**Published:** 1989

**Authors:** H. M. Roder, R. A. Perkins

**Affiliations:** National Institute of Standards and Technology, Boulder, CO 80303

**Keywords:** fluid, platinum, pressure, resistance, thermal conductivity, transient hot-wire

## Abstract

The resistance of metals is a function of applied pressure, and this dependence is large enough to be significant in the calibration of transient hot-wire thermal conductivity instruments. We recommend that for the highest possible accuracy, the instrument’s hot wires should be calibrated in situ. If this is not possible, we recommend that a value of *γ*, the relative resistance change with pressure, of −2×10^−5^ MPa^−1^ be used to account for the pressure dependence of the platinum wire’s resistance.

## 1. Introduction

During the last decade the transient hot-wire has evolved as the primary method for the measurement of thermal conductivity of fluids. In these systems, the wire, usually platinum, is used both as the heating element and as the temperature sensor. The primary variable measured is the change of resistance of the wire as a function of time. The wire is immeirsed directly in the fluid, and any pressure experienced by the fluid is transmitted to the wire. It is well known that the resistance of metals changes with applied pressure (see, for example, ref. [1]). What is perhaps not widely appreciated is that this effect is substantial enough to be detected at the relatively low fluid pressures encountered in the typical transient hot-wire measurement of thermal conductivity. In this paper we report resistance measurements on 12.5 *μ*m diameter platinum wires as a function of pressure up to 70 MPa.

## 2. Method

In the transient hot-wire method the resistance-temperature relation of the platinum wires must be defined accurately to achieve reliable results for thermal conductivity measurements. In our versions of this method [2,3] we have opted for a wire calibration in situ. In both the low-temperature version, 70 to 300 K, [2] and the high-temperature version, 300 to 600 K, [3] we use a Wheatstone bridge to measure resistances. Compensation for end effects is provided by placing the long hot wire in one working arm of the bridge and a shorter, compensating, wire in the other. In contrast to most other instruments where times are measured at a null voltage point, in our instruments the voltages developed in the bridge are measured directly as a function of time with a fast digital voltmeter.

Before making each thermal conductivity measurement, the bridge is balanced using a small supply voltage, 50 to 100 mV, to give an output as near to zero as possible. We have one calibrated standard resistor in each side of the bridge. The voltage drops measured across the standard resistors yield the currents in each side of the bridge. Resistances are then determined in terms of voltage drops across the elements of the bridge, that is the hot wires, the leads, and the adjustable balancing resistors. The hot-wire resistances measured during the balancing of the bridge, together with the cell temperatures determined from the calibrated platinum resistance thermometer mounted on the cell, are taken as the in situ calibration of the wires.

As described in reference [2] the resistance relation for each wire was represented by an analytical function of the type,

$$R(T,P)=A+BT+CT^{2}+DP,$$ (1)

where *R*(*T,P*) is the wire resistance, *T* is the temperature, and *P* is the applied pressure. The pressure dependence was small, but statistically significant. In the low-temperature system [2] the high-pressure cell closure can accommodate only three leads. These leads are the two current leads and one potential tap at the corner of the bridge. Additional potential taps are placed outside the high-pressure cell. Since there are still short sections of the leads within the cell we cannot measure the voltage drops across each hot wire directly. The leads are steel and copper, and were accounted for by using resistance tables and by measuring the length and diameter of each piece. For the low-temperature system [2] we could not be certain that the observed pressure depedence resulted only from the platinum wire since other explanations were also possible.

## 3. Apparatus

During the last three years we have modified and improved the low-temperature system to enable us to measure the thermal diffusivity of the fluid at the same time that we measure the thermal conductivity. The motivation to measure the thermal diffusivity is, of course, to obtain values of the specific heat, *Cp. A* description of the changes in the system and initial results on argon are given in [4,5]. Most of the changes made to the apparatus improved the measurement of resistance. The theory of the measurement of thermal conductivity by the transient hot-wire method has been given in [6]. For the measurement of thermal diffusivity, the corrections required by the theory had to be evaluated anew [7]. It turned out, not unexpectedly, that accurate measurement of the wire resistance was of the utmost importance.

**Figure 1:**
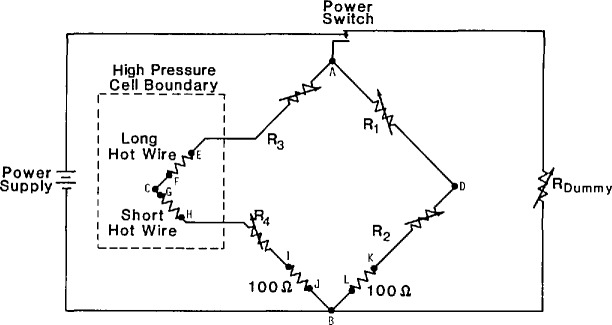
*A* schematic circuit diagram of the Wheatstone Bridge. Potential taps are indicated by the points A-L.

All of the changes and improvements were also incorporated into our second apparatus, which was designed to operate at higher temperatures [3]. The Wheatstone bridge circuit, shown in figure 1, was changed to improve the accuracy with which the hot-wire resistances and the initial balance condition could be measured. This was accomplished by adding a digital voltmeter to the system capable of measuring voltages to 0.5 *μ*V at the 200 mV level. The voltages required in the wire calibration and bridge-balancing cycle are fed to the voltmeter through a new multiplexer. Each arm of the new bridge is about 200 Ω at ambient temperature and includes a series of precise decade resistances. Because the arms have higher resistances than in the old system, it is possible to include a calibrated 100 Ω standard resistor in each side of the bridge; thus the current in each side of the bridge can be measured independently.

**Figure 2:**
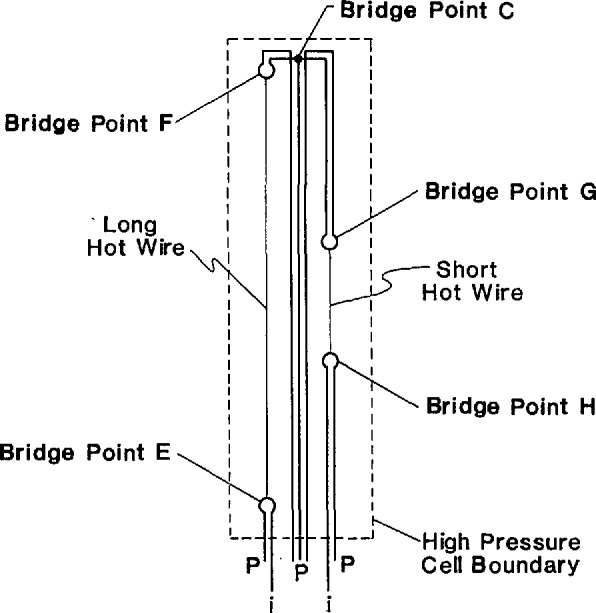
Arrangement of current leads (i) and potential taps (P) within the high pressure cell. Bridge points correspond to those in figure 1.

The new high-temperature apparatus differs in several other aspects from the low-temperature one. Important to the present discussion is the fact that in the new system there are seven leads into the cell rather than three. With the new arrangement of the leads, shown in figure 2, it is now possible to measure the voltages across both the long and the short hot wires directly. This eliminates the need to account for (nuisance) lead resistances and their dependence on temperature within the cell. The resistance measurements are made as follows. With a supply voltage between 50 and 100 mV the current in the left side of the bridge (fig. 1) is determined by measuring the voltage drop across the calibrated 100 Ω standard resistor at potential taps I and J. The voltage drops across the hot wires are measured between voltage taps E and F, and G and H. The taps are shown in figure 1 while the physical arrangement is shown in figure 2. In summary, we can now measure each resistance with an uncertainty of 9 mΩ which is considerably better than the uncertainty of the earliest version of the low-temperature instrument. The measurements described here for nitrogen at 300 K with pressures up to 70 MPa are the first to be made with the new high-temperature system.

## 4. Measurements

**Figure 3:**
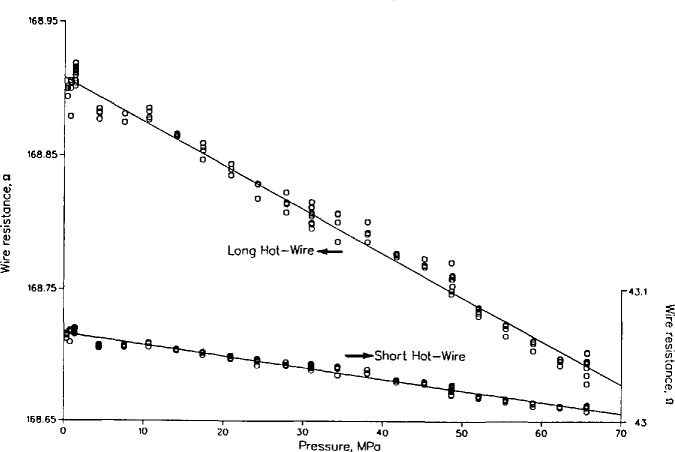
Wire resistance for long and short hot wires as a function of pressure for a temperature of 300 **K.**

Figure 3 shows the measured resistances of both the long and short hot wires as a function of the fluid pressure up to 70 MPa at 300 K. The resistance of each wire clearly decreases as the pressure increases. We represent the data with a straight line for each wire,

$$\begin{array}{l} R(T,P)=R(T,0)+DP,\text{or}\\ R(T,P)/R(T,0)=1+\gamma P. \end{array}$$ (2)

The resistance lines are shown in figure 3; the coefficients and standard deviations are:
*R*(300.0)*D*St. dev.(1 σ)*γ*ΩΩMPa^−1^ΩMPa^−1^long hot wire168.9085−0.003 294 460.009−1.95×10^−5^long hot wire43.0667−0.000 870 630.003−2.02×10^−5^

In order to compare our results with those of Bridgman [1], we compare the ratios *R*(300,70)/*R*(300,0). We obtained a ratio of 0.9986_4_ for the long hot wire and 0.9985_9_ for the short hot wire. The value interpolated from Bridgman’s paper [1] is 0.998_6_. The agreement with Bridgman’s value is excellent, and we conclude that the measured resistance changes are caused by changes in the fluid pressure. These resistance changes might also be temperature dependent. From the wire calibration established during recent thermal conductivity measurements on nitrogen [8], which were, however, made in the low-temperature system, we obtained values of *γ* of −1.85×10^−5^ for 250 K and −2.2×10^−5^ for 100 K, a change of about 20 percent in *γ.*

## 5. Summary

We close with the recommendation that, if the ultimate in accuracy is to be obtained in a thermal conductivity measurement, an in situ calibration of the hot wires should be performed. If an in situ calibration of the hot wires cannot be performed, then the resistance change of the wires can be taken as an additive, calculated correction using a *γ* of −2×10^−5^ MPa^−1^, as shown in eq (2).
